# Insights into Organochlorine Pesticides Exposure in the Development of Cardiovascular Diseases: A Systematic Review

**DOI:** 10.34172/aim.2023.86

**Published:** 2023-10-01

**Authors:** Mohammad Ali Mohammadkhani, Soraya Shahrzad, Mehrdad Haghighi, Reza Ghanbari, Ashraf Mohamadkhani

**Affiliations:** ^1^Department of Electrical Engineering, Technical and Vocational University, Tehran, Iran; ^2^Department of Cardiology, Shariati Hospital, School of Medicine, Tehran University of Medical Sciences, Tehran, Iran; ^3^Infectious Diseases and Tropical Medicine Research Center, Shahid Beheshti University of Medical Sciences, Tehran, Iran; ^4^Gene Therapy Research Center, Digestive Diseases Research Institute, Tehran University of Medical Sciences, Tehran, Iran; ^5^Liver and Pancreatobiliary Diseases Research Center, Digestive Diseases Research Institute, Shariati Hospital, Tehran University of Medical Sciences, Tehran, Iran

**Keywords:** Cardiovascular disease, Exposure, Organochlorine pesticide, Polychlorinated biphenyls

## Abstract

Many human diseases such as cancer, neurological diseases, autism and diabetes are associated with exposure to pesticides, especially organochlorine pesticides. However, pesticide exposure is also associated with cardiovascular disease (CVD) as the leading cause of death worldwide. In this systematic review, results on the link between organochlorine pesticide pollution and CVD were collected from databases (Medline (PubMed), Scopus and Science Direct) in May 2022 from studies published between 2010 and 2022. A total of 24 articles were selected for this systematic review. Sixteen articles were extracted by reviewers using a standardized form that included cross-sectional, cohort, and ecological studies that reported exposure to organochlorine pesticides in association with increased CVD risk. In addition, eight articles covering molecular mechanisms organochlorine pesticides and polychlorinated biphenyls (PCBs) on cardiovascular effects were retrieved for detailed evaluation. Based on the findings of the study, it seems elevated circulating levels of organochlorine pesticides and PCBs increase the risk of coronary heart disease, especially in early life exposure to these pesticides and especially in men. Changes in the regulatory function of peroxisome proliferator-activated γ receptor (PPARγ), reduction of paroxonase activity (PON1), epigenetic changes of histone through induction of reactive oxygen species, vascular endothelial inflammation with miR-expression 126 and miR-31, increased collagen synthesis enzymes in the extracellular matrix and left ventricular hypertrophy (LVH) and fibrosis are mechanisms by which PCBs increase the risk of CVD. According to this systematic review, organochlorine pesticide exposure is associated with increased risk of CVD and CVD mortality through the atherogenic and inflammatory molecular mechanism involving fatty acid and glucose metabolism.

## Introduction

 Pesticides are a group of components used for pest control in agricultural areas and in public health strategies for the purpose of protecting plants and humans. Insecticides, a variety of pesticides to kill insects and herbicides, as inhibitors of unwanted plant growth, are also classified as pesticides. With wider applications, they also include products such as biocides, which are designed to control pests and disease vectors.^1^ Most of these chemicals are designed to disrupt the physiological activities of the target organism, leading to dysfunction and shortened life, although some of them are considered dangerous to humans.^2,3^ Their widespread use is a primary ecological concern as their residues can be a significant source of environmental and food chain contamination.^4,5^ A large group of functional pesticides included organochlorines, organophosphates and carbamates. Data from various pesticide assessments show that organochlorine pesticides and polychlorinated biphenyls (PCBs) are ubiquitously detected in the environment.^6,7^ Due to the low cost and the need to fight against various pests, these pesticides are most widely used in the developing countries of Asia, which can often be absorbed and accumulated in the fatty tissues of crustaceans and saltwater fish, and thus enter the human food chain.^4,8,9^

 Increased use of pesticides primarily in agriculture and community health is associated with increased risks of human exposure to pesticides accompanying various human diseases, including cancer, neurodegenerative diseases, autism, diabetes, obesity and cardiovascular disease (CVD).^3,10-12^

 High exposure to pesticides causes oxidative cellular damage stress and metabolic disorder such as hyperlipidemia and, in the long term, CVD.^10,13^ For example, higher prevalence rates of diabetes and circulatory system diseases and coronary heart disease are found in people who work in factories producing herbicides and chlorophenol.^14^ The mechanism of toxicity of pesticides and their destructive effects on CVD have been proven by some experimental studies.^15,16^

 Owing to the restrictions imposed on the use of organochlorine pesticides and PCBs, it seems that their environmental levels are generally decreasing; however, early-life exposure cases who are at risk of disease are significant. Therefore, this review has gathered and summarized the previous studies in the overall evidence on exposure to organochlorine pesticides in relation to higher risk of CVD and the molecular mechanisms involved in toxicity and CVDs in exposure to organochlorine pesticides.

## Materials and Methods

 A comprehensive search on the association of organochlorine pesticides and PCBs and CVD was conducted through a bibliographic review on Medline (PubMed) (1354 articles), Scopus and ScienceDirect (459 articles) in May 2022, using studies published between 2010 and 2022. The keywords used to search the related texts included “organochlorines pesticide exposure”, “cardiovascular disease”, “polychlorinated biphenyls” and also “cardiovascular disease organochlorines pesticide mechanism”. In addition, the search for sources and citations of selected studies was completed. Restrictions were set for studies published in English.

 Two researchers independently screened the titles and abstracts of all identified studies to find potentially relevant articles. The studies were considered qualified if they met all of the following inclusion criteria: 1) Studies that evaluated the relationship of organochlorine pesticides and PCBs with the incidence of CVDs or the establishment of risk factors leading to CVDs; and 2) studies that addressed the molecular mechanism of the cardiovascular effects of organochlorines pesticide and PCBs.

 The exclusion criteria were: 1) studies whose full texts were not available; 2) studies published in languages other than English or Persian; 3) duplicate publications; and 4) studies with insufficient data.

**Figure 1:**
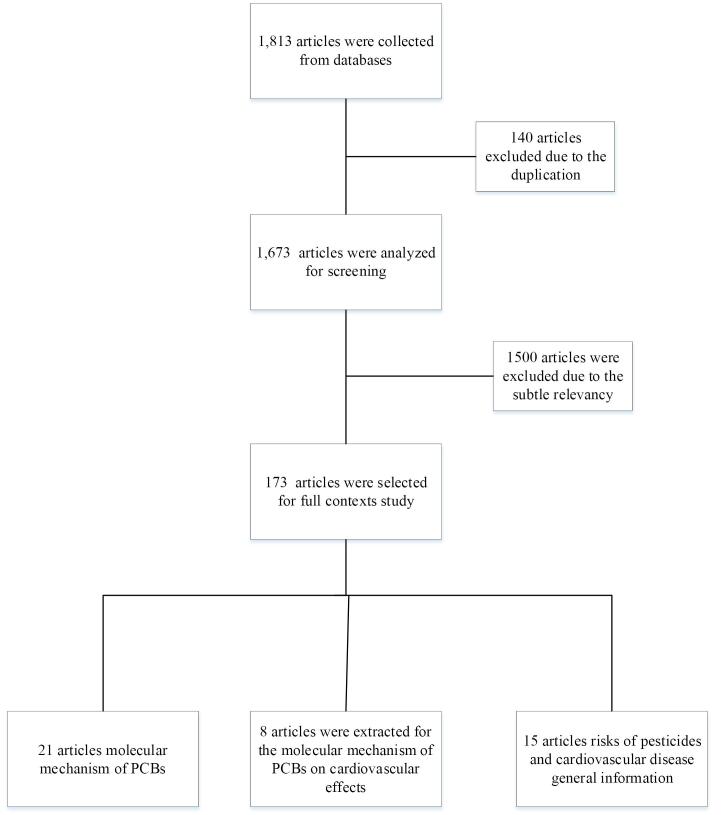
Study Selection on Exposure to Organochlorine Pesticides in the Development of Cardiovascular Disease and the Molecular Mechanisms of the effect of Pesticide Toxicity in Humans

**Figure 2:**
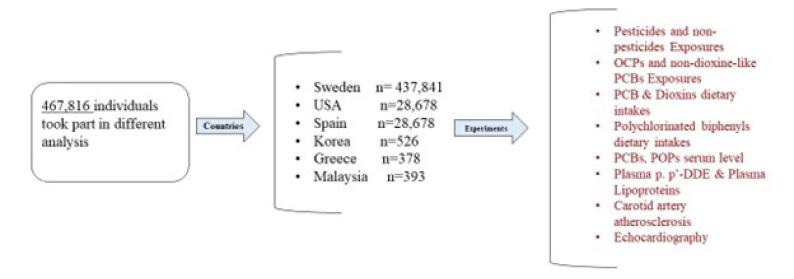
Studies Investigating the effects of Organochlorine Pesticides and PCBs in Different Countries and the Number of People Involved in the Experiments

Figure 1 shows the selected articles on the relationship between exposure to organochlorine pesticides and PCBs and CVDs and also the molecular mechanisms of the effect of pesticide toxicity in humans. The various studies conducted to investigate the effects of organochlorine pesticides and PCBs on the human body in different countries and the number of people participating in different experiments are shown in Figure 2.

## Results

 Among 1813 article retrieved, 140 were duplicates, and 1500 articles were excluded for lack of relevance to the subject. Afterwards, 173 studies published up to January 2022 that assessed the risk of CVD from exposure to organic chlorine-based pesticides were thoroughly examined. Sixteen articles were selected by reviewers using a standard form inclosing overall study information (title, first author’s name, year of publication, place of study).

 In addition, of the 21 articles on the molecular mechanism of organochlorine pesticides and PCBs identified from the literature search, eight articles including cross-sectional, cohort, and ecological studies covering molecular mechanisms on cardiovascular effects were retrieved for detailed evaluation (Figure 1).

## Toxic Effects of Organic Chlorine-Based Pesticides on Living Organisms

 Persistent organic pollutants (POPs) are carbon-based organic substances that may be produced intentionally or unintentionally and are an important human health problem due to their persistence, ubiquity, and bioaccumulation. They are roughly divided into two groups of fat-soluble and non-fat-soluble chemicals. POPs are commonly detected in humans, although their use has been banned in most developed countries since the 1970s because of their potential negative impact on human health.^17,18^

 Organochlorine pesticides and PCBs, as common POPs, also defined as endocrine disrupting chemicals (EDCs), are lipophilic chemicals that contain at least one covalently bonded chlorine atom; the number of chlorine atoms is related to its half-life. PCBs, consisting of 10 homologues and lot numbers of structures of the same type with different numbers and positions of chlorine atoms, are commonly used in transformers as well as in industrial materials such as colored paper, plastics and paints.

 Organochlorine pesticides (OCPs) include a wide range of chemicals, including dichlorodiphenyltrichloroethanes (DDTs), as well as their metabolites (especially dichlorodiphenyldichloroethylene or DDE) known as dioxins, that are a family of toxic compounds and harmful to health with similar structure and properties. DDT is still needed and used for vector control because there is no alternative of equivalent efficacy and operational feasibility, especially in areas with high transmission of vector-borne diseases.^19,20^

 Organochlorine insecticides have been already successfully used to control malaria and typhoid, but residues from past use and their accumulation in the environment have been reported, especially in the soil as well as fatty tissues of wild and domestic animals, and marine sediments and fish.^8,17,21^

 The main ways of pesticides entering the body are ingestion in food or water, inhalation, and absorption through the skin and through the eyes, which is known as non-occupational exposure.^22^ Diet is a significant source of exposure, particularly for infants and children. Also, PCBs and organochlorine pesticides readily cross the placenta and breast milk, resulting in early life exposure via maternal transfer. There is also evidence of metabolism of DDT to DDD by members of the human gut microbiota (e.g. *Eubacterium limosum*).^21^

## Exposure to Organochlorine Pesticides and PCBs Related to the Risk of Cardiovascular Disease

 People with high levels of PCBs and DDT in their blood have been found to be at higher risk for various human disease. The main effects of organochlorine pesticides are stimulation of the central nervous system through interference with various ion channels, although carcinogenicity and teratogenicity are also important features.^19^ However, exposure to pesticides during human life is clearly associated with plaque formation in the carotid artery.^23,24^ Regular exposure to pesticides can lead to myocardial infarction, congestive heart failure, stroke, arrhythmia and sudden death.^14,25,26^ Chronic exposure to organic chlorides through accumulation in the adipose tissue potentially activates mechanisms such as inflammation and oxidative stress in the development of CVD.^27^ Studies have shown that the accumulation of organic chlorine in the adipose tissue is associated with hypertension, elevated serum lipids, and obesity and metabolic syndrome, which are proven risk factors for CVD.^28^

 Consumption of foods containing PCBs in sources such as fatty fish has been considered as a risk factor for heart failure, although this risk has been corrected by receiving eicosapentaenoic acid (EPA) and docosahexaenoic acid (DHA).^25^ Fish is generally the main source of n-3 long-chain polyunsaturated fatty acids EPA and DHA; however, when consumed in diet, PCB exposure should also be considered.^27^ Unlike PCBs, taking EPA-DHA is associated with a moderate reduction in resting heart rate and blood pressure.^23^ Therefore, the cardioprotective properties of EPA-DHA can counteract the PCB-induced inflammation of endothelial cells.^25^

 In addition, the association of POPs with type 2 diabetes (T2D) has been identified, although the mechanisms involved are still under investigation.^29^ Fat-soluble POPs, like organochlorine pesticides, are transported by lipoproteins and accumulate in the adipose tissue. Cross-sectional studies have also shown an association between high levels of these compounds in the bloodstream and high levels of low-density cholesterol, triglycerides, and lipoproteins.^30^ On the other hand, fat-insoluble POPs, such as perfluoroalkyl compounds (PFAS), are also associated with elevated total cholesterol levels. High levels of predominantly chlorinated PCBs and fluorinated PFAS have been shown to be associated with carotid artery atherosclerosis, but only in women.^31^ Both cross-sectional and prospective studies have shown that dioxins, PCBs, and PFAS are associated with CVD and mortality.

## Overview of the Relationship Between Organochlorine Pesticides and CVD as well as the Risk Factors of CVD Development

 There is ample evidence to support the view that exposure to various chemicals belonging to POPs can be associated with lipid abnormalities, carotid atherosclerosis, myocardial infarction, stroke and CVD.^30^ Despite the association between POP and T2D, epidemiological support for this association is not comprehensive; however, the evidence suggests that a person’s body fat history may influence the association between POP and T2D.^29^ As mentioned, PCBs are highly lipid-compatible compounds that are biologically stored in the food chain and thus, affect the human body’s metabolism during growth. The effect of POPs on metabolic diseases such as T2D and obesity was evaluated by Donat-Vargas et al and they showed that dietary PCB intake, estimated using the food frequency questionnaire, was associated with a higher prevalence of obesity. However, more longitudinal studies are needed to confirm the results.^32^

 Accordingly, a 15-year longitudinal analysis in the adult GraMo group, focusing on exposure to persistent organic pollutants, showed that concentrations of most pollutants trend positively with increasing average consumption of CVD medication.^33^ There is no doubt that there is a positive relationship between PCB exposure and hypertension. Previous studies in different populations and individuals exposed to POP early in life have reported a positive association between serum concentrations of PCBs or similar structures and the incidence of hypertension.^28,34,35^ In a Mediterranean cohort study, dietary PCB intake, assessed by a food frequency questionnaire, indicated a higher risk of developing hypertension during follow-up.^28,35^

 Agricultural studies have also shown that exposure to pesticides is associated with increased blood pressure and elevated serum lipoproteins, which are known to be risk factors for CVD.^36^ The relationship between circulating levels of POPs and blood pressure in a population-based sample of men and women was also examined in the Prospective Investigation of the Vasculature in Uppsala Seniors (PIVUS) study. One hundred and three POPs were analyzed in this study. The results showed that high levels of circulating p, p’-DDE were associated with hypertension.^37^ In a cohort case study to evaluate the association between levels of POPs and stroke risk, 526 members and 111 strokes from the Korean Cancer Prevention Study-II were evaluated. The results showed that increased serum levels of POPs were associated with increased risk of stroke, particularly ischemic stroke.^38^ In a cross-sectional study, the National Health and Nutrition Examination Survey 1999–2002 assessed serum POPs such as polychlorinated dibenzo-p-dioxins (PCDDs), polychlorinated dibenzofurans (PCDFs), dioxin-like PCBs, dioxin-unlike PCBs and organochlorine pesticides in 524 adult participants over 40 years of age recently diagnosed with hypertension. Obviously, organochlorine pesticides were not associated with hypertension in both gender. Indeed, the serum concentrations of some POPs were associated with hypertension in a sex-specific manner. Among women, serum concentrations of PCDDs and PCDFs were associated with recent hypertension, but not in men, while PCBs, conversely, tended to be positively associated with hypertension only in men.^39^ PCBs have been also shown to increase the risk of myocardial infarction in men.^27^

 Although a number of general population studies have shown that vascular endothelial function is impaired by exposure to dioxins and PCBs, leading to progressive atherosclerosis, men with pre-existing coronary heart disease with exposure to PCBs, but not dioxins, exhibited further subclinical atherosclerosis. Exposure to PCBs appears to increase the risk of coronary artery disease in men from an early stage.^26^ To investigate the association of some POPs with atherosclerosis, PFASs, a group of POPs, were evaluated in the PIVUS study. The presence of carotid artery atherosclerosis was examined by ultrasound in 1016 patients with 70 years of age and by testing for eight plasma PFASs in more than 75% of these participants. In this cross-sectional study, a marked gender difference was observed among some PFASs, particularly long-chain PFUnDA, with atherosclerotic markers in women.^31^ In order to evaluate the effect of chronic exposure to mixed pesticides on the increased risk of CVD, 198 Malay men exposed to pesticides and 195 Malay male workers not exposed to pesticides were studied. Data were collected through a questionnaire, blood analysis and CVD evaluation. The results of the study showed that chronic exposure to a mixture of pesticides among workers involved in mosquito control may be associated with depression and increased ox-LDL and heart rate.^40^

 In addition to hypertension, diabetes and obesity, POPs can also lead to left ventricular hypertrophy (LVH). In a prospective study to evaluate the effect of POPs on LVH, the Vasculature in Uppsala Seniors (PIVUS), left ventricular mass index, relative wall thickness, and geometric groups of LVH were assessed by echocardiography. Also, 21 POPs were measured in 1,016 people aged 70 years. The results of the study showed that circulating POPs were associated with increased left ventricular wall thickness and concentric left ventricular regeneration, independent of LVH risk factors, indicating the role of this environmental contaminant in abnormal growth of the left ventricle.^41^

 Fine particles of agricultural chemicals potentially cause peripheral arterial disease or peripheral vascular disease. Progression of peripheral vascular disease is the result of narrowing of the arteries of the heart or brain with very fine particles or fatty acids, which can eventually manifest as infarction or tissue death or coronary artery disease.^42-44^ Obstruction of arteries is due to the penetration of very small particles that can easily get stuck in the bloodstream, resulting in atherosclerosis.^14^ The disease is associated with increased cardiovascular morbidity and mortality in most countries. Organochlorine pesticides have been also shown to be involved in the development of peripheral arterial disease.^45^ Occupational exposure to pesticides in a group of 7,557 Japanese-American men from the Kuakini Honolulu Heart Program also showed a positive association between the incidence of CVD and age-related pesticide exposure.^14^

 A quick overview of the articles evaluating the harmful effects of organochlorines, including chlorinated dioxins, PCBs and other dioxin-like compounds in terms of CVDs or the risk factors that lead to CVD is detailed in Table 1.

**Table 1:** Selected Articles on the Relationship between Exposure to Pesticides and Cardiovascular Diseases

**Country, References **	**Exposure to Organochlorine Pesticides**	**Effects **
Spain, Miguel Perez-Carrascosa F^33^	Exposure to 5 OCPs and 3 non-dioxin-like PCBs was estimated in 387 participants from the GraMo adult cohort by analysis of their adipose tissue concentrations at recruitment.	POP exposure signifies a risk factor for CVD.
Sweden, Tornevi A^29^	Plasma concentrations of chlorinated persistent organic pollutants were assessed in 129 case-controls from longitudinal population-based data of northern Sweden.	The association between evaluated plasma POPs and T2D which could be influenced by body fat
Spain, Donat-Vargas C^32^	Dietary PCB intake was assessed using a semi-quantitative food frequency questionnaire in 12 313 participants without obesity at baseline who were followed for a median of 8.1 years.	Higher incidence of obesity during follow-up
Spain, Donat-Vargas C^35^	Dietary intake of PCBs was assessed at baseline with 8.3 years of follow-up in 14 521 participants of the Seguimiento Universidad de Navarra project as Spanish cohort through validated 136-item semiquantitative food frequency questionnaire.	Higher risk of developing hypertension
Spain, Donat-Vargas C^28^	Plasma dioxin-like and non-dioxin-like PCBs were evaluated in 850 participants for 10 years in a subcohort in the Northern Swedish health and disease study.	Hypertension
USA, Goncharov A^34^	Serum PCBs, 35 individual PCB congeners, and nine chlorinated pesticides were evaluated in 394 Anniston residents who were not taking antihypertensive medications.	Serum concentrations of PCBs, PCB congeners with multiple ortho chlorines, were firmly associated with systolic and diastolic blood pressure.
Sweden, Jugan J^36^	Plasma p, p'-DDE and plasma lipoproteins were assessed in 57175-year-old Swedes in the PIVUS cohort who were not prescribed lipid-lowering drugs.	High p, p'-DDE interacts with lipoproteins within plasma and plays a role in CVD.
Sweden, Lind PM^37^	Twenty-three POPs were analyzed in 1016 subjects aged 70 years in the PIVUS study.	Circulating p, p'-DDE levels were associated with developing hypertension.
Korean, Lim JE^38^	Serum POPs levels were assessed in 526 members and 111 stroke cases from the Korean Cancer Prevention Study-II.	PCBs were positively associated with lipid abnormalities and carotid atherosclerosis with increased risk of ischemic stroke.
Sweden, Bergkvist C^27^	Dietary exposure to PCBs adjusted for known cardiovascular risk factors, long-chain omega-3 fatty acids (eicosapentaenoic and DHAs) and methyl mercury exposure were assessed from the population-based Swedish Cohort of Men for 12 years of follow-up (433,243 person-years). 3005 incident cases of myocardial infarction (654 fatal) were found.	In adjusted models, increased risk of myocardial infarction in men was associated with dietary PCB exposure.
Spain, Donat-Vargas C^26^	Coronary artery calcium score and dietary exposure to PCBs and dioxins were examined in 1844 men aged 50 and without CVD, who participated in the Aragon Workers' Health Study.	Dietary exposure to PCBs, but not dioxins, was linked to higher prevalence of coronary calcium and subclinical coronary atherosclerosis in middle-aged men.
Sweden, Lind PM^31^	Carotid artery atherosclerosis was assessed by ultrasound in 1016 subjects 70 years of age and older in the PIVUS study. Eight PFAS were also identified in approximately 75% of the participants' plasma by UPLC-MS/MS.	The association of PFUnDA with long chain, and atherosclerosis markers with more pronounced relationships was observed in women.
Malaysia, Samsuddin N^40^	Exposure to mixed pesticides and the risk of cardiovascular disease (CVD) were evaluated in 198 male Malaysian workers exposed to pesticides and 195 non-exposed Malaysian males.	Chronic exposure to mix-pesticide among workers involved in mosquito control was an independent predictor of brachial and aortic systolic/diastolic blood pressure.
South Africa, Sekhotha MM^45^	To study the relationship between pesticide particles and CVDs in agricultural industries based on existing data.	There is a close relationship between agrochemicals particles and the development of myocardial infarction, CHF, stroke, arrhythmia, and sudden death.
Honolulu, Berg ZK^14^	A cohort of 7,557 Japanese-American men from the Kuakini Honolulu Heart Program were evaluated for various levels of occupational pesticide exposure over 10 years of follow-up.	A positive association was perceived between the age-adjusted incidence of CVD and high levels of exposure to pesticides.
Sweden,Sjoberg Lind Y^41^	Echocardiography and 21 POPs were measured in 1016 individuals aged 70 years in the PIVUS.	LVH

OCPs, Organochlorine pesticides; PCB, polychlorinated biphenyl POP, Persistent organic pollutant CVD, cardiovascular disease; PIVUS, Prospective Investigation of the Vasculature of Uppsala Seniors; CHF, congestive heart failure; LVH, Left ventricular hypertrophy; DHA, docosahexaenoic acid; T2D, type 2 diabetes; UPLC–MS/MS, ultra-performance liquid chromatography–tandem mass spectrometry.

## Molecular Mechanism of Toxicity of Organochlorines Pesticides Regarding CVD

 It has been shown that low doses of organochlorine such as PCB126 interfere with the normal metabolic function of the heart and cause diseases such as heart failure, hypertension and cardiomyopathy.^23,24^ Nonetheless, PCBs or PCB congeners are directly involved in increasing the synthesis of cholesterol and triglycerides which could accumulate in the myocardium, which is a clear sign of cardiac hypertrophy.^46^ Exposure to low-dose PCB126 disrupts cardiac energy metabolism by altering the regulatory function of the peroxisome proliferator-activated γ receptor (PPARγ) gene.^47,48^ PPARs increase insulin sensitivity, mitochondrial function and the metabolism of fatty acids and glucose, as well as exert anti-inflammatory and anti-atherogenic properties on vascular walls and immune cells.^15^ During heart failure, the mechanism of ATP production changes with the increasing rate of glucose uptake and glycolysis resulting in cardiac hypertrophy and myocardial fibrosis.^16^

 Impaired metabolism of glucose and lipids in the liver by PCB126 also results in insufficient hepatic homeostasis that contributes to cardiovascular inefficiency.^49^ Examination of the metabolic effects of organochlorine pesticides on CVD showed that chronic exposure to PCBs leads to detrimental effects on carbohydrate metabolism, although the amino-acids glycine and threonine, which are also found in collagen and elastin, are increased. Mice exposed to PCB126 have been shown to increase collagen synthesis enzymes, thereby increasing the proteins required for extracellular matrix and fibrosis.^48^

 PCBs and organochlorine pesticides are capable of reducing paraoxonase (PON1), the lipophilic antioxidant factor of high-density lipoprotein (HDL) cholesterol. Increased POPs concentrations in HDL in CVD patients is accompanied by decreased PON1 activity which may impair HDL function to reduce LDL susceptibility to lipid peroxidation.^43^

 The mechanism of action of DDT and DDE isomers (collectively called DDx) as risk factors for cardiovascular dysfunction has already been described by Truong et al. They suggested that DDx engaged both proteins Ryanodine receptor type 2 (RyR2), with function of releasing calcium-induced calcium ions from sarcoplasmic reticulum (SR), and SR/ER Ca^2+^ ATPase (SERCA2a) with the role of Ca^2+^ reabsorption in SR, in the cardiomyocyte to accelerate the heartbeat.^50^ Cardiotoxicity of DDT and its metabolites interacting with RyR2 and SERCA2a has also been reported in tropical regions.^51,52^

 Congenital heart disease (CHD) is also a multifactorial disease; nevertheless, its association with environmental contaminants such as PCBs has been demonstrated in numerous epidemiological and experimental studies. The effects of contact with environmental chemicals that lead to CHD in humans are similar to the effects of these compounds on vertebrates such as fish, which are more sensitive to these compounds. Therefore, the similarity of the human fetal heart in the third week of pregnancy with the zebrafish, the freshwater fish belonging to the Cyprinid family, allows to evaluate these special effects.^53^ Extraordinary uptake of PCB126 in zebrafish disrupts the permeability of endothelial cells via the NO signaling pathway. The binding of PCB126 to the AHR receptor activates the metabolism of xenobiotics, leading to the formation of reactive oxygen species and oxidative stress at low concentrations of PCB126.^53,54^ In studying the toxic effects of PCBs and dioxin-like compounds such as AhR agonists, it has been shown that continuous activation of AhR by dioxins increases blood pressure and cardiac hypertrophy, induces atherosclerosis and the formation of aortic aneurysms abdominal muscles and impairs vascular relaxation. However, PCB126-induced cellular oxidative stress is likely to be modulated by vitamin E.^55^

 In addition, PCBs participate in epigenetic alterations of histones by inducing reactive oxygen species and also stimulating vascular endothelial inflammation by modifying the expression of miRNAs such as miR-126 and miR-31.^56^ Consecutive cellular oxidative stress leads to an imbalance in the antioxidant state and activity of inflammatory genes mediated by PCBs and endothelial cell dysfunction.^57^ TNF-alpha, C-reactive protein, and the inflammatory biomarker IL-6 could be crucial indicators of heart failure. Additionally, recent population-based studies have revealed that PCBs can control telomere length (TL) and telomerase activity. The opposite link between PCBs and TL in lymphocytes and buccal cells suggests that POPs have distinct opposite effects on TL, hTERT activity, and telomere-related repair gene expression.^58^

## Conclusion

 The widespread exposure of humans to a wide range of organic chlorines such as PCBs, dioxins and DDT, mainly affecting the metabolic and inflammatory pathways of cells and increasing the atherogenic index, leads to the development of risk factors of CVD such as increased blood pressure, obesity and T2D and as a result, death from CVDs. More research is needed on the incidence and mortality of CVD in populations exposed to pesticides, particularly organochlorine pesticides.
